# Management of Occupational Exposure to Engineered Nanoparticles Through a Chance-Constrained Nonlinear Programming Approach

**DOI:** 10.3390/ijerph10041231

**Published:** 2013-03-26

**Authors:** Zhi Chen, Yuan Yuan, Shu-Shen Zhang, Yu Chen, Feng-Lin Yang

**Affiliations:** 1 Department of Building, Civil, and Environmental Engineering, Concordia University, Montreal, QC H3G 1M8, Canada; E-Mail: yuany@encs.concordia.ca; 2 Laboratory of Industrial Ecology and Environmental Engineering (MOE), School of Environmental Science and Technology, Dalian University of Technology, Dalian 116024, China; E-Mails: zhangss@dlut.edu.cn (S.-S.Z.); chenyu@dlut.edu.cn (Y.C.); yangfl@dlut.edu.cn (F.-L.Y.)

**Keywords:** manufactured nanomaterials (MNMs), occupational exposure, environmental health and safety standards (EHS), emerging risk management, nonlinear programming, chance-constrained programming, uncertainty analysis

## Abstract

Critical environmental and human health concerns are associated with the rapidly growing fields of nanotechnology and manufactured nanomaterials (MNMs). The main risk arises from occupational exposure via chronic inhalation of nanoparticles. This research presents a chance-constrained nonlinear programming (CCNLP) optimization approach, which is developed to maximize the nanaomaterial production and minimize the risks of workplace exposure to MNMs. The CCNLP method integrates nonlinear programming (NLP) and chance-constrained programming (CCP), and handles uncertainties associated with both the nanomaterial production and workplace exposure control. The CCNLP method was examined through a single-walled carbon nanotube (SWNT) manufacturing process. The study results provide optimal production strategies and alternatives. It reveal that a high control measure guarantees that environmental health and safety (EHS) standards regulations are met, while a lower control level leads to increased risk of violating EHS regulations. The CCNLP optimization approach is a decision support tool for the optimization of the increasing MNMS manufacturing with workplace safety constraints under uncertainties.

## 1. Introduction

Manufactured nanomaterials (MNMs) are man-made particles having at least one dimension of roughly 1–100 nm [1]. MNMs have been employed in a wide spectrum of industrial sectors in recent years, including energy, medicine, electronics, environmental protection, cosmetics, food, agriculture and many other areas. This is due to their unique properties, such as small size and associated large surface area to mass ratio, increased surface reactivity, and altered physico-chemical properties [2,3]. The unique chemical and physical properties of MNMs have raised issues regarding occupational health and safety (EHS) in manufacturing facilities [4], particularly when handled in large amounts [5]. MNMs can be released to the occupational atmosphere during industries producing processes, where MNMs are synthesized, purified, and packaged, thereby becoming commercial products. As a result, MNMs can enter worker’s body through inhalation, skin contact and ingestion during manufacturing [6]. Recent publications indicate that chronic occupational exposure to MNMs may lead to a number of negative health and reproductive problems, including hepatic injury [7], genotoxicity [8,9], carcinogenicity [10], cytotoxicity (apoptosis) and risks of cardiovascular diseases [11,12], and reproductive damage [13,14]. 

So far, relatively few publications have directly approached modelling MNMs occupational exposure risks, which include Monte Carlo models that compare various levels of environmental health and safety (EHS) standards for single wall carbon nanotube (SWCNT) manufacturing [15], and expert opinions on development of exposure-response functions for nanomaterials [16]. To minimize the risks of MNMs to workers’ health as well as to maximize their economic benefits, optimization models are efficient tools to manage MNMs manufacturing processes and reduce occupational exposure. Usually, optimization algorithms involve geometric programming, dynamic programming, and linear programming methods [17]. However, most MNMS-producing processes are complex systems with inherent nonlinearities, where the systems are best described by nonlinear optimization method [18].

Previously, nonlinear programming (NLP) has been widely employed in technological optimization of manufacturing processes, such as agriculture [16], electronics industry [19,20], and construction [21]. NLP is one of the most frequently applied operational algorithms for real world problems as its fundamental theories have been well studied and as a result, a wide spectrum of user-friendly solution software with powerful computational capabilities have been developed. One limitation of NLP is that it relies heavily on the inherent assumption that all relevant variables have deterministic values [22].

Unfortunately most real-life problems involve a certain amount of uncertainties making the implementation of NLP a difficult task [23]. For instance, work-related exposures to MNMs are associated with a number of uncertainties in relation to control options and risk quantification. Uncertain variables for a nonlinear MNMS manufacturing system analysis may include: (1) MNMS workplace release data. Previous studies indicated a pressing need to distinguish background nanoparticles’ concentrations, process-generated nanoparticles’ concentrations and MNMs in workplace risk assessment [24]; (2) Occupational exposure limits: it may take several years to establish human no-effect levels (NELs) for each kind of MNM, so in many cases no nano-specific occupational health and safety standards are available [25]; and (3) MNM occupational exposure control efficiency and control cost. Data on efficiency and cost of MNM control methods are vague [15]. It is seen that the above-mentioned uncertainties have not been well quantified in the previous studies. 

Considering uncertainties in the optimization, application of chance-constrained programming (CCP) has been reported to environmental management problem [26]. CCP is an effective way to deal with various uncertainties, where uncertain parameters are considered random variables and described using probability density functions. CCP can be used to convert a stochastic programming model into an equivalent deterministic model, and also to incorporate other uncertain optimization methods, such as fuzzy mathematical programming, within the nonlinear programming general framework [27]. Therefore, combining CCP with NLP is an approach that could be used to deal with various uncertainties pertaining to MNM and workplace exposure control.

The objectives of this study are: (1) development of a nonlinear optimization approach for modeling the MNM manufacturing process towards a maximum nanomaterial production at a minimum cost of workplace exposure control under a number of constraints; (2) development of chance-constrained nonlinear programming (CCNLP) through an integration of NLP and CCP to address the system uncertainties including the randomness of exposure data; and (3) examination of the developed model to a single-wall carbon nanotube (SWNT) manufacturing process. 

## 2. Methodology

### 2.1. The Nanotechnology Manufacturing Process

There are two main engineering design methods for preparing nanoparticles, top-down and bottom-up. The top-down approach works on the basis of breaking down a large piece of material into smaller pieces, and in the case of nanostructures these dimensions are in the nanometer range: 1 to 100 nm. The bottom-up approach relies on increasing the size of small molecules or atoms up to the size of MNMs via techniques such as nucleation, self-assembly and evaporation. Generally, bottom-up techniques are less waste-producing than top-down techniques. It is often suggested that bottom-up techniques should be the ultimate tools for sustainable manufacturing, as they allow for customized design of reactions and processes at the molecular level, thereby minimizing waste [28]. Synthesized MNMs should be processed further, e.g., to be purified, inspected, packaged, and then becoming commercial products. Here, we take the single-walled nanotube (SWNT) production process as our example.

#### High-Pressure Carbon Monoxide (HiPco) Process for Manufacturing Single-Walled Nanotubes (SWNTs)

Single-walled nanotubes (SWNTs) are cylindrical molecules of graphite with diameters of 1 to 2 nm that have attracted considerable interest due to their superior electrical, mechanical, and thermal properties, and particularly, their fascinating ability to withstand high current density (109 Amps/cm^2^) [29,30]. The use of SWNTs has raised concerns because of their resemblance to asbestos in terms of dimensions, rigidity and solubility, as these factors determine fiber toxicity leading to lung fibrosis, so consequently carbon nanotubes known as high aspect ratio nanoparticles (HARN) have engendered concern about their potential for a similar risk as that from the asbestos [31]. 

**Figure 1:**
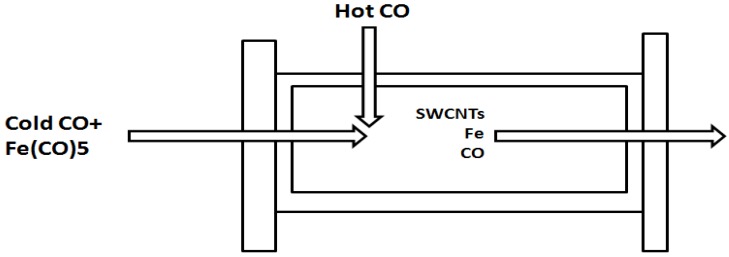
Schematic diagram of the HiPco synthesis process.

Among the several methods available for producing carbon nanotubes, three technical processes are commonly used: arc ablation (arc), chemical vapor deposition (CVD) and high-pressure carbon monoxide (HiPco). Because the HiPco process is significantly less costly ($450/g *vs.* $1,830/g and $1,586/g for arc ablation and CVD, respectively) [16], we focused on the HiPco manufacturing method in the CCNLP model to explore profits under various EHS standards (High, Medium, Low). In the HiPco process, it is proposed that iron clusters form first, then solid carbon nucleates and grows SWNTs. Iron pentacarbonyl (Fe(CO)_5_) is injected into a stream of CO gas at high temperature (800–1,000 °C) and pressure (≥10 atm). The iron clusters form by aggregation of iron atoms from the decomposition of Fe(CO)_5_ via Equation (1) (see below) around 250 °C. There are two main functions for the iron clusters. They act as catalysts for carbon source decomposition as well as SWNT formation sites. The clusters grow by collision with additional metal atoms and other clusters, eventually reaching a diameter comparable to that of a SWNT, 0.7–1.4 nm, corresponding to 50–200 iron atoms. By the time they reach this size, CO can disproportionate (a specific type of redox reaction) on the surface of such cluster via the Boudouard reaction (Equation (2)) to yield solid carbon, and SWNTs will nucleate and grow from these clusters [32]. Figure 1 shows the material flows in the manufacturing reactor. The SWNTs and iron particles pass through the reactor propelled by the hot, dense gas flow, and into the product collection apparatus. The CO gas recalculates back through the gas flow system and reactor using a compressor. The product contains Fe particles and other by-products and requires subsequent purification [33,34].

Fe(CO)_5_ →→ Fe + 5CO (1)

2CO(g) →→ C(s) + CO_2_(g) (2)

### 2.2. Optimization for Occupational Exposure Risk Management of SWNT Manufacturing

#### 2.2.1. The Nonlinear Programming (NLP) Approach for Occupational Exposure Risks Management of SWNT Manufacturing

As one of the most important tools of optimization, nonlinear programming (NLP), which is a type of deterministic optimization, is implemented here to manage nanoparticles exposure risks in the workplace. It is an objective function which is subject to a set of linear constraints, where the objective function is nonlinear. For a given nano-manufacturing plant, the general form of this optimization approach is written as follows:

Objective function = (1) – (2) – (3)

where:

(1) = profits from nanoparticle manufacturing per year;
(2) = production costs of nanoparticles per year;
(3) = exposure control costs per year.

Constraints include:

(a) mass balance constraints;
(b) production volume constraints;
(c) occupational exposure limit constraints.

The model below was developed using the optimization method to evaluate cost and exposure control trade-offs of the SWNT manufacturing process:

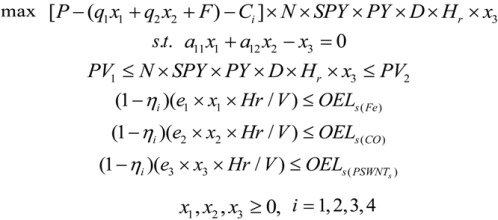
(3)
where *x*_1_, *x*_2_ are the feed rate of Fe(CO)_5_ and CO (in g/h), respectively; x_3_ is the SWNT material production rate (g/hr); P is the revenue from each gram of SWNT manufactured ($/g); q_1_, q_2_ is the cost of Fe(CO)_5_ and CO for each gram of SWCNT produced, respectively ($/g); F is the total costs of SWCNT except for the raw materials ($/g; the raw materials are included in the above parameters); C_i_ is the exposure control cost of SWCNT per gram produced ($/g) in every scenario; N is the number of production lines; *Hr* is the working hours per day (hours/day); SPY is the synthesized product (carbon nanotube) yield (%); *PY* is the SWCNT purification yield (%); D is the working days per year (days/year); a_11_, a_12_ are the percentages of Fe(CO)_5_ and CO used to synthesize SWCNTs, respectively (%); PV_1_ and PV_2_ are the minimum and maximum production volume of SWCNTS per year (g/yr); *η_i_* is the removal efficiency of MNMs emissions at each control levels (%); *e*_1_, *e*_2_, *e*_3_ represent the emission coefficients of nano-sized Fe, CO and SWCNTs, respectively (They are used to quantify the emission of nano-sized Fe, CO and SWCNTs from a unit production of SWNTs and are calculated ); *V* is the volume of the workplace (m^3^); OELs_(Fe)_, OELs_(CO)_, OELs_(SWNTs)_ are the occupational exposure limits for iron powder, CO and SWNTs, respectively (mg/m^3^). 

The value of the objective junction [*P* –(*q*_1_*x*_1_ + *q*_2_*x*_2_ + *F*) - *C_i_*] × *N* × *SPY* × *PY* × *D* × *H_r_* × *x*_3_ is the annual net profits of SWCNT manufacturing; (*q*_1_*x*_1_ + *q*_2_*x*_2_) represents the cost of the two raw materials used for each gram of SWCNTs produced; F includes the expense of direct labour, energy, equipment, installation, tools, building and fixed overhead [35]; *N* × *SPY* × *PY* × *D* × *H_r_* × *x*_3_ is the annual production volume of SWCNTs; *q*_1_, *q*_2_ are the cost of Fe(CO)_5_ and CO for each gram of SWCNTs produced, respectively. 

The production volume is the number of manufacturing (production) lines multiplied by the throughput for a single line. And the annual throughput rate of one HiPco synthesis production line is calculated as: *Throughput* = *SPY* × *PY* × *D* × *H_r_* × *x*_3_, where the SWCNT synthesis product yield represents the relative amount of carbon nanotubes (single-wall carbon nanotubes and multi-wall carbon nanotubes) expected from the converted carbon; and the purification yield indicates the percent of SWCNT removed from the carbon product compared to the total SWNT created from the synthesis step. 

Five constraints are material flow balance, annual production volume and cumulative exposure to three hazardous materials, which will be explained as follows: (1) *Material flow balance*. From Equations (1) and (2), we know that SWNT is synthesized from the carbon elements of CO and Fe(CO)_5_, e.g., 10 moles of CO (or Fe(CO)_5_) produce 5 moles of SWNT and 5 moles CO_2_. (2) *Annual production volume*. The production volume should within a certain range [36]. (3) *The emissions of nano-sized Fe*, *CO and SWNTs* should be less than the allowable amount in relation to their occupational exposure limits. 

#### 2.2.2. Chance-Constrained Nonlinear Programming (CCNLP) Calculations for Occupational Exposure Risk Management of SWNT Manufacturing

**Figure 2:**
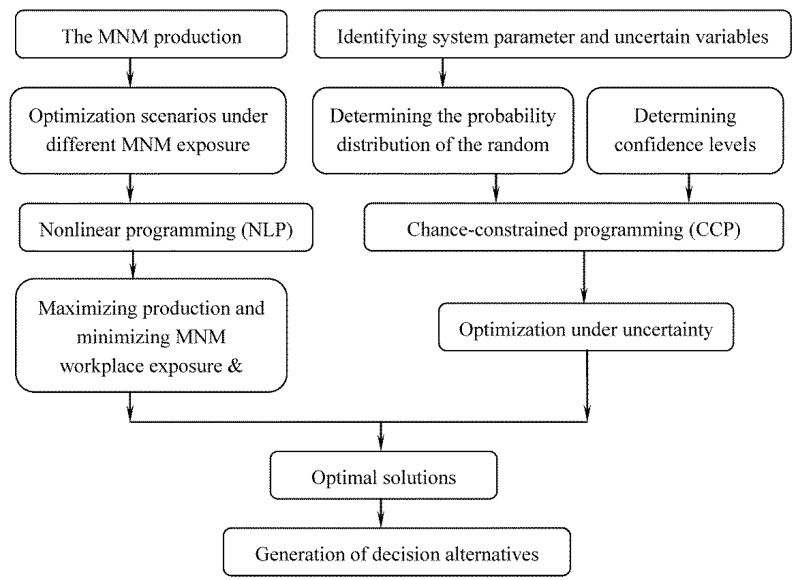
Framework of the CCNLP optimization method.

Chance-constrained programming (CCP) is a typical stochastic programming model for risk-based decision making. The CCP model maximizes the objective function subject to constraints with specified predetermined confidence levels, where these confidence levels are provided as appropriate safety margins by the decision-makers. The CCP model provides information on the trade-offs between the objective function’s tolerance values of the constraints, and the prescribed level of probability, which could be valuable to decision makers. A mathematical program with chance-constrained parameters is presented as follows:

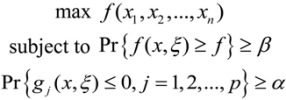
(4)
where x is an n-dimensional decision vector, ξ is a stochastic vector, *f*(*x*,*ξ*) is the return function, and *g_j_*(*x*,*ξ*) are stochastic constraint functions, *j* = 1, 2, …, *p*, Pr { ∙ } denotes the probability of the event in { ∙ }, and *α*, *β* are predetermined confidence levels of the constraint and objective, respectively. The detailed model formulated using this optimization approach for our Texas factor case study is described in the next section. In the single-wall carbon nanotube exposure control case study discussed above, emission coefficients of the theee pollutants (*i.e*., nano-sized Fe, CO and SWCNTs) were uncertain. A: emission coefficient can be calculated as follows [37]:
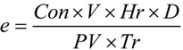
(5)
where Con is the estimated concentration of pollutant (μg/m^3^·h or mg/m^3^·h); V is the volume of the workplace (m^3^); Hr is the working hours per day (h/day); D is the working days per year (days/year); PV is the average production volume of SWCNTS per year (g/yr); Tr is the transformation factor (1,000,000 when the unit for Con is μg/m^3^·h and 1,000 if the unit is mg/m^3^·h. For a specific MNM manufacturing section, V, Hr, D, PV and Tr are deterministic values. Con is an uncertain variable which can be presented as a probability density function. Thus, emission coefficient (*e*) also can be described by a probability density function. Assuming these emission coefficients contain random variables, then the model can be rewritten as:

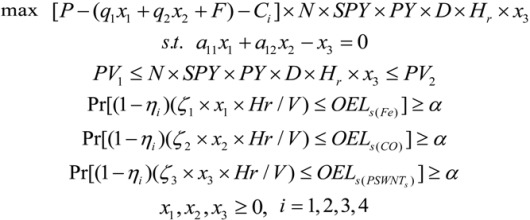
(6)
where *ζ*_1_, *ζ*_2_, and *ζ*_3_ , which are functions containing random variables, replace *e*_1_, *e*_2_, *e*_3_ to represent emission coefficients of nano-sized Fe, CO and SWCNTs, respectively. Pr { ∙ } means that the cumulative exposure should be less than the “no observable effect” level(NOEL) ≥ *α* of the time. Figure 2 shows a framework of the CCNLP optimization method. 

## 3. Case Study

### 3.1. Overview of the Case Study

A study case was adapted from a SWNT manufacturing plant located in Houston, TX, USA [35], where nano-specific occupational environmental health and safety (EHS) standards were voluntarily implemented. In the plant, the HiPco method is used to produce 87% pure SWNT. There are nine HiPco synthesis lines in one production room with a size of 30 m × 20 m × 3 m. The plant operates eight hrs/day and 365 days/year. Each line produces SWNTs with 97% synthesis product yield and 90% purification yield. During the production, three air pollutants are emitted that workers are exposed to: SWNTs, nano-size iron powder and carbon monoxide. 

**Table 1:** Summary of environmental health and safety (EHS) levels and the related exposure control measures (adapted from [14]).

	**EHS levels**		
Type of EHS Control	Low	Medium	High
Engineering Controls			
General exhaust-ventilation	24 h, 28.31 m^2^ ventilation rate, $10,000 capital cost, $ 3,000/year operating cost	24 h, 28.31 m^2^ ventilation rate, $10,000 capital cost, $ 3,000/year operating cost	24 h, 28.31 m^2^ ventilation rate, $10,000 capital cost, $ 3,000/year operating cost
Fume hoods		$4,000 capital cost for 0.58 m^2^ equipment and $9,500 for 2.3 m^2^ equipment	$4,000 capital cost for 0.58 m^2^ equipment and $9,500 for 2.3 m^2^ equipment
Enclosure of processes			50% decrease in labor productivity, 50% extra equipment cost
Administrative controls			
Annual worker training	8 h of training, $560/year instructor cost	8 h of training, $560/year instructor cost	8 h of training, $560/year instructor cost
Air monitoring	Monthly monitoring, $20,000/equipment capital cost	Weekly monitoring, $20,000/equipment capital cost	Biweekly monitoring, $20,000/equipment capital cost
Medical monitoring			$950/worker/year

### 3.2. The NLP and CCNLP MNM Workplace Model Information and Scenarios

As shown in Table 1, 4 levels of EHS standards were used in our model (None, Low, Medium and High). This are defined to represent the possible strategies under which nano-EHS standards might be imposed [38]. Control costs and reduced manufacturing efficiency possible at each EHS control level are defined in Table 2. 

**Table 2:** Assumed control cost and reduced efficiency for each levels of control (adapted from [15]).

Control Level	Cost ($/g)	Reduced Efficiency (η)
No	0.00	0.0
Low	10.00	0.1
Medium	78.00	0.5
High	210.00	0.8

We assume that the emission coefficients of nano-sized Fe, CO and SWCNTs are normally distributed random variables with known means and standard deviations (Table 3). For the chance-constrained programming of SWNT exposure, the predetermined confidence levels in four scenarios were tested at 90%, 95% and 99% levels, respectively.

**Table 3:** Mean and standard deviations of the emission coefficients of nano-sized Fe, CO and SWCNT.

	Mean	SD	Reference
nano-sized Fe (ζ_1_)	0.00135	0.00070	(Calculated from [25])
CO (ζ_2_)	0.03700	0.00040	(Calculated from [39])
SWCNT (ζ_3_)	0.00287	0.00227	(Calculated from [25])

The main assumptions of this model include that: (1) the manufacturing reactions are conducted under the conditions of 1,050 °C and 30 *atm*; (2) the reactions reach the dynamic balance very quickly; (3) no other source of Fe, CO and SWCNT pollution exists in the workplace; (4) the concentrations of Fe, CO and SWCNT in the air of the manufacturing room are homogeneous, and then no agglomeration is considered; (5) workers do not have access to safety clothing and respirators. The values of parameters used based on references are given in Table 4. 

**Table 4:** Summary of Parameters for NLP and CCNLP methods.

Symbols	Units	Definition	Values	Reference
P	$/g	price of SWCNT	1,000.00	[36]
q_1_	$/g	cost of Fe(CO)_5_ per gram SWCNT produced	0.2	[33]
q_2_	$/g	cost of CO per gram SWCNT produced	37.0	[34]
F	$/g	total cost of SWCNT other than the raw material	411.3	(calculated from [36])
N	\	number of production lines	9	[36]
SPY	%	synthesis product yield	97	[36]
PY	%	purification yield	90	[36]
D	days/year	working days per year	365	[36]
Hr	hours/day	working hours per day	8	[36]
a_11_	%	% Fe(CO)_5_ used to synthesize SWCNT	15.0	(calculated from [32])
a_12_	%	% CO used to synthesize SWCNT	21.0	(calculated from [32])
PV_1_	g/yr	minimum production volume of SWCNT per year	0.0	[36]
PV_2_	g/yr	maximum production volume of SWCNTS per year	20,000	[36]
e_1_	\	emission coefficient of nano-sized Fe	0.003	[5]
e_2_	\	emission coefficient of CO	0.037	(calculated from [40])
e_3_	\	emission coefficient of SWCNT	0.005	[5]
OEL_(Fe)_	μg/m^3^	occupational exposure (OE) limit for nano-sized Fe and Fe(CO)_5_	7.9	[41,42]
OEL_(CO)_	mg/m^3^	OE limitfor CO	40.0	[43]
OELs_(SWNTs)_	μg/m^3^	OE limit for SWCNT	7.0	[42]

## 4. Results and Results Analysis

### 4.1. Results of the Nonlinear Programming (NLP) Calculations

**Figure 3:**
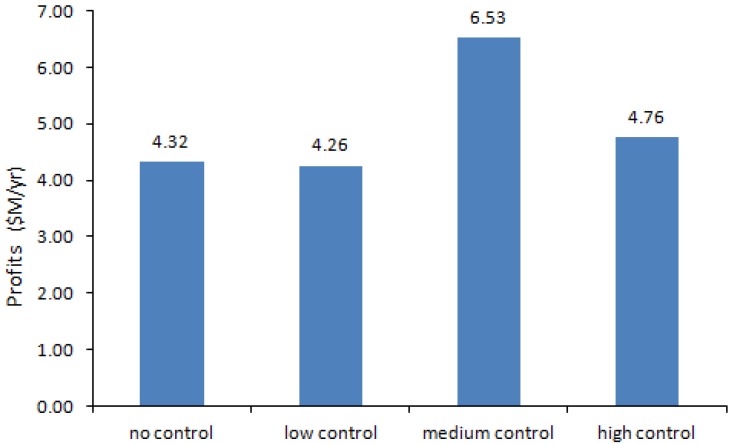
The annual net profits calculated from NLP method.

**Figure 4:**
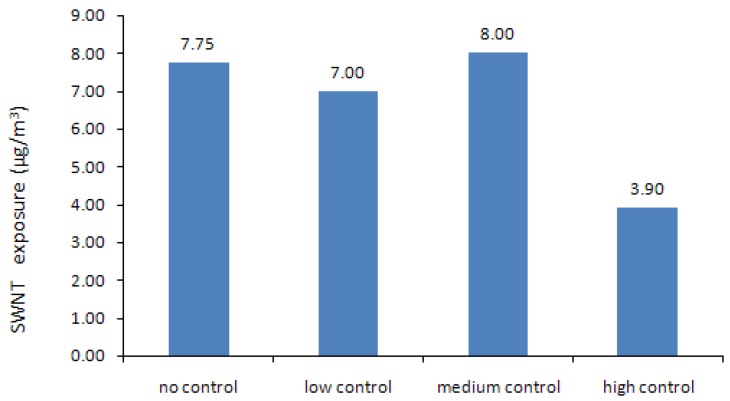
SWNT exposure results from NLP method.

As Figure 3 shows, the total annual net profits for no control, low control, medium control and high control are $4.32 M/yr, $4.26 M/yr, $6.53 M/yr and $4.76 M/yr, respectively. The corresponding worker occupational exposures to SWNTs are 7.75, 7.02, 7.97, and 3.87 μg/m^3^ (Figure 4). The results suggest that SWNTs are the major threat to workers’ health, compared to CO and nano-Fe, because in the no, low and medium control scenarios, their concentrations are higher than the occupational exposure limit (OEL) [39,44]. Table 5 describes the production volumes, production costs, annual net profits, and atmospheric concentrations of SWNT, nano-Fe and CO in the factory under four different management scenarios, where the production volumes, costs, profits and concentrations are obtained from the nonlinear programming (NLP) calculations. 

**Table 5:** Results of the NLP method.

Control Level	Production Volume (g/yr)	Production Cost ($/g)	Profit ($M/yr)	SWNT Exposure (μg/m^3^)	CO Exposure (μg/m^3^)	Fe Exposure (μg/m^3^)
No	8,003	459.94	4.32	7.75	213.14	1.00
Low	8,049	460.30	4.26	7.02	209.46	0.90
Medium	16,452	524.83	6.53	7.97	218.21	0.50
High	20,000	552.08	4.76	3.87	135.64	0.20

If no any protection strategies are adopted, the optimum solution is obtained, in which the profit is $4.32 M/yr when production volume is 8,003 g/yr, production cost is 459.94 $/yr and SWNT concentration is 7.75 μg/m^3^ (1.11 higher than the OEL). The no-control scenario is set as the base line. 

When a low level control option is taken, the 10% particle removal efficiency only slightly (0.5%) raises the production volume, while the total cost (production cost plus control cost) increase 2%. As a result, the released SWNT concentration decreases to 7.02 μg/m^3^ (1.003 higher than the OEL) and the profit decreases 14%. 

In the medium control scenario, the production volume increase significantly (106%) due to the 50% removal efficiency, and then the SWNT exposure and profit reach the highest level: 7.97 μg/m^3^ (1.15 higher than the OEL) and $6.53 M/yr, although the total cost increases by 31%. 

When a high level of protection is implemented, the production volume would reach the maximum 20,000 g/yr (150%), and the total cost would also increase significantly (66%), which generates the second highest profit of $4.76 M/yr. Its 90% removal efficiency decreases the SWNT exposure to 45% below the OEL. 

The results indicate that, under the high control option, the significant limiting constraint is not the OEL but the MNMs production constraint. The maximum production volume of this study plant is 20,000 g/yr due to the limited space, labor and investment. Thus, although the OELs allow much higher production volume in the high control scenario, only 20,000 g SWCNT could be produced per year as shown in the results. Low production and high control efficiency (90%) lead to a low concentration of emitted pollutants. For Fe exposure, exposure in medium control option is almost twice of the high control level, like SWCNT and CO. For profits of high control level, the control cost at 210.00 $/g is much higher than medium control level at 78.00 $/g, low production and high control cost lead to low profits. 

In general, the SWNT exposure concentrations and profits increase with the rise of the manufacturing production volume caused by the stricter standards and reach the maximum when the medium level control option is adopted. Nevertheless, they decrease later because the effects of increasing production volume have been neutralized by the effects of the rise of remove efficiencies (total costs). 

### 4.2. Results of the Chance-Constrained Nonlinear Programming (CCNLP) Model

**Figure 5:**
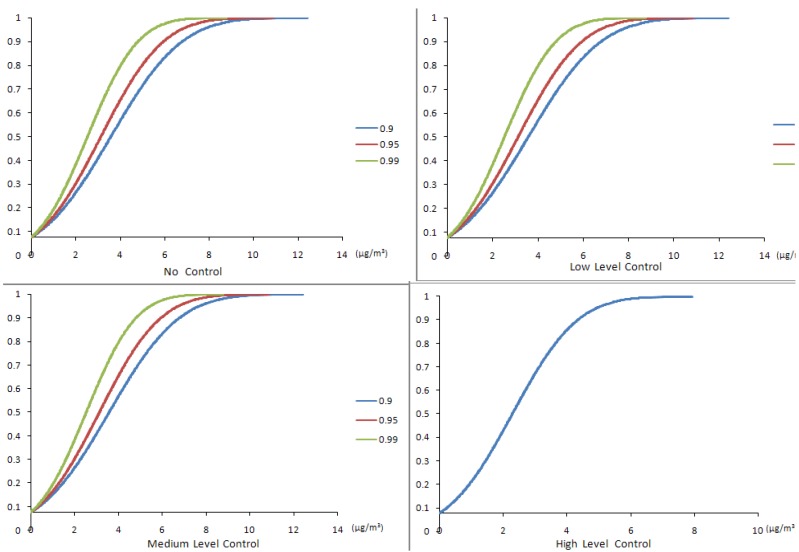
Cumulative probability distributions of SWNT exposure results from CCP method.

**Figure 6:**
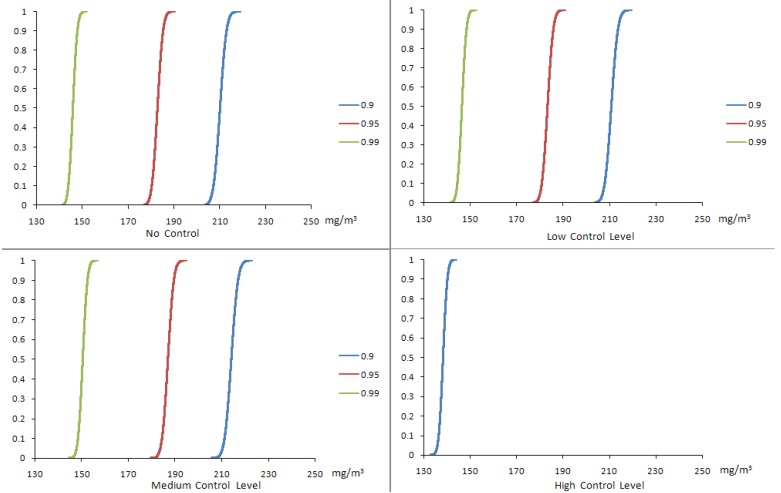
Cumulative probability distributions of CO exposure results from CCP method.

**Figure 7:**
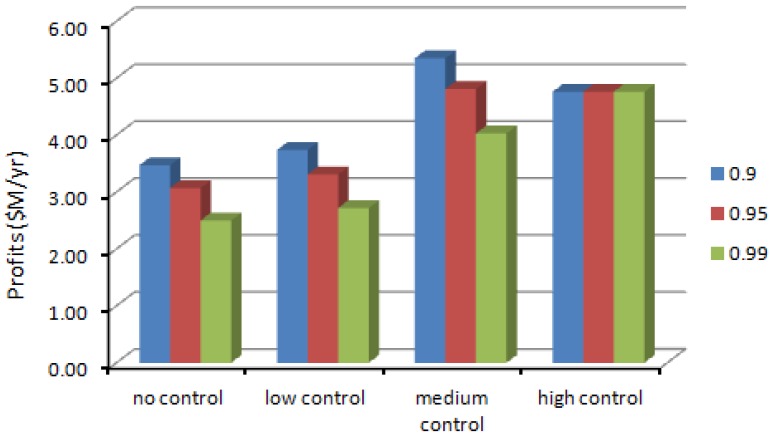
The annual net profits results from CCNLP method.

Figure 5, Figure 6 show the worker exposure ranges of SWNT and CO under different confidence α_i_ levels and Figure 7 shows the corresponding SWNT manufacturing profits obtained using the CCP calculations. Table 6 describes the CCP model calculated results for SWNT production volume, production costs, net profits and estimated worker exposure ranges for the three air pollutants (SWNT, nano-Fe and CO) at different confidence levels. In general, the α_i_ levels represent a set of probabilities at which the constraints can be violated (*i.e*., the admissible risk of violating the constraints). Therefore, the relation between the profits and the confidence levels demonstrates a trade-off between production volume and control constraint-violation risk. A decreasing α level means a decreasing limitation for the OEL constraints, which may then result in an increased production volume. The increased production volume would potentially increase the profits and risks of violating the EHS standards. A lower α level brings on a higher profits but a higher risk of violating the EHS constraints; meanwhile, a higher α level results in a lower profits but an increased reliability of satisfying the occupational standards. These alternatives represent a compromise between economic benefit and environmental health and safety (EHS) requirements. 

**Table 6:** Results of the CCNLP method.

Confidence Level	Control Level	Production Volume (g/yr)	Production Cost ($/g)	Profit ($M/yr)	SWNT Exposure (μg/m^3^)	CO Exposure (μg/m^3^)	Fe Exposure (μg/m^3^)
0.9	No	6,263	446.59	3.47	0.00–12.42	201.85–218.72	0.00–1.60
	Low	6,952	451.87	3.74	0.00–12.41	202.52–219.44	0.00–1.60
	Medium	12,504	494.51	5.35	0.00–12.40	205.74–222.93	0.00–1.60
	High	20,000	552.08	4.76	0.00–7.90	132.74–143.84	0.00–1.02
0.95	No	5,483	440.60	3.07	0.00–10.88	202.52–219.44	0.00–1.40
	Low	6,080	445.18	3.31	0.00–10.85	176.13–190.85	0.00–1.40
	Medium	10,944	482.53	4.81	0.00–10.85	179.66–194.05	0.00–1.40
	High	20,000	552.08	4.76	0.00–7.90	132.74–143.84	0.00–1.40
0.99	No	4,428	432.49	2.51	0.00–8.78	140.13–151.84	0.00–1.13
	Low	4,910	436.19	2.72	0.00–8.76	140.70–152.45	0.00–1.13
	Medium	8,856	466.49	4.03	0.00–8.78	144.59–156.67	0.00–1.13
	High	20,000	552.08	4.76	0.00–7.90	132.74–143.84	0.00–1.13

## 5. Discussion

### 5.1. Comparison between the Modeling Results and Literature Data

NLP and CCNLP models also serve as cost models for the HiPco SWNT manufacturing process. Results from these two models are compared with data of the earlier cost model [40]. For the NLP model, the production cost range is from 459.94 to 552.08 $/g when the production volume is from 8,003 to 20,000 g/yr, and Isaacs *et al.* [36] reported a range of production costs from 440 to 510 $/g for the same production volume and a HiPco manufacturing process. It is seen that results from the NLP and previous cost analysis are close to the literature data (with a maximum 8% difference).

For the CCNLP model, Table 6 gives a production cost range from 440.60 to 552.08 $/g when production volume is from 5,483 to 20,000 g/yr, and previous cost model gives a range from 440.60 to 552.08 $/g under the same conditions. It is seen that the CCNLP model and Isaacs *et al.* [36] SWNT production cost model also share the same range and trend under the same conditions (with a maximum 8% difference). 

### 5.2. Comparison of the NLP and CCNLP Models

NLP is an easier-to-use optimization method than CCNLP when all parameters have deterministic values, where the results are expressed by point values. As an extension of NLP method, CCNLP is not only an optimization approach, but also an uncertainty estimation method to handle random values, whose results are expressed as ranges. Results from the nonlinear programming (NLP) SWNT model and chance-constrained nonlinear programming (CCNLP) SWNT model indicate that they can provide alternative risk-benefit management schemes in the engineered nanoparticle production process. For instance, in the NLP model, a desire to acquire the highest economic benefit will run the risk of violating occupational health and safety standards, when the control level is medium (the NLP model predicts if profits are $6.53 M/yr, then SWNT emission will be 1.14 higher than the suggested safety levels). Willingness to compromise and accept a 27% lower but sill reasonably high economic benefit (in this case $4.76 M/yr of profits at the higher level of occupational health and safety control with the lowest worker SWNT exposure, which is 3.90 μg/m^3^) will guarantee satisfactory air quality requirements, and fewer employee health issues. 

The CCNLP model predicts that if profits reach maximum value, which is $5.35 M/yr, with the medium level of EHS control with 0.90 confidence level, the SWNT pollutant emission could be 1.8 higher than the recommended occupational exposure limit. To reduce SWNT pollutant level to the minimum (the high control level with 0.99 confidence level), the model suggests that profits must be reduced by 11% (low to $4.76 M/yr). Since workers’ MNMs-related diseases would result in large class-action lawsuits cost hundreds of millions in the future caused by chronic overdosed exposure to MNMs, the model predict that it is economically better to comply with the recommended EHS guidelines rather than violate it [45]. 

When decision makers chose different levels of MNM emissions, the trade-off between profits and violation risks can be analyzed by the NLP and CCNLP model. And, results from both models demonstrate that voluntary implementation of the high level of EHS protection can lead to reduce MNMs exposure risks with insignificant decrease in profits.

The advantages of the chance-constrained nonlinear programming (CCNLP) optimization are: (1) it can be used to simulate the relationship between MNM production rate and worker occupational exposure level in a manufacturing settings based on limited information; (2) it can be used as a MNM quantitative exposure estimation model to predict the range of exposure concentrations possible in the workplace under a given set of conditions; (3) it is a good tool for decision makers to help analyze trade-offs between manufacturing revenue and the risk of violating environmental health and safety (EHS) standards. For example, as discussed above, if short term (about 20 years) maximum profit is prioritized, the medium level of EHS control with 0.90 confidence level may be the best choice, but if worker’s health is prioritized, managers may choose the high control level with 0.99 confidence level. 

NLP and CCNLP model results also indicate that a higher level EHS control and higher stringent enforcement lead to a lower probability of EHS constraint violation and a lower manufacturing profitability and *vice versa*, and implementation of the highest level of EHS standards and most stringent enforcement with lower economic profits would be the optimal solution in the long run, by examining the relationships between economic benefits and security (the occupational exposure limits-violation risk).

### 5.3. Uncertainty and Sensitivity

This study has provided a modeling tool to optimize the MNMs manufacturing process with main the sources of uncertaintities being quantified, thus to minimize environmental concerns associated with nanotechnology industry. In a real world application, OEL selection and other sources of uncertainties should be scrutinized. For example, in this study as the resulted exposure concentration of nano-sized Fe (and Fe(CO)_5_) is at a very low level compared to OEL_s(Fe)_ [39], also significantly lower than other nano-sized species, as shown in Table 5, sensitivity analysis of model runs has proved that variation of OEL_s(Fe)_ does not change the modeling results. Also, it is unlikely that the MNMs will be homogeneously diluted in the manufacturing room, fate and transport of MNM in the environment including its agglomeration effects can be studied [46]. 

## 6. Conclusions

In this research, a chance-constrained nonlinear programming (CCNLP) approach is developed for modeling and planning of nanomaterial manufacturing under an acceptable workplace exposure risk scenario. In the CCNLP, methods of chance constrained programming and nonlinear programming are combined within an optimization framework to effectively reflect uncertainties that present in different formats. In particular, the developed CCNLP model allows dynamic trade-off analyses of objectives from different stakeholders.

NLP and the CCNLP model results indicate that a higher level of EHS control and more stringent enforcement lead to a lower probability of EHS constraint violations and a lower manufacturing production and *vice versa*, and implementation of the highest level of EHS standards and most stringent enforcement with lower economic profits would be the optimal solution in the long run, considering the relationships between economic and exposure risk control priorities. The advantages of the proposed chance-constrained nonlinear programming (CCNLP) model are: (1) it can be used to quantify the dynamic relationship between MNM production rate and worker occupational exposure level in manufacturing settings with practically limited information; (2) it could serve as an MNM exposure estimation model to predict the range of exposure concentrations possible in the workplace under given manufacturing conditions; (3) it is an effective tool for decision makers to help analyze trade-offs between manufacturing revenue and the risk of violating environmental health and safety (EHS) standards. For example, as aforementioned, if short term (about 20 years) maximum profit is prioritized, the medium level of EHS control with 0.90 confidence level may be a good decision point, but if the workplace exposure risk is of high concern, we may choose the high risk control level with 0.99 confidence level. As a new extension of mathematical programming methods for dealing with system uncertainties, the developed CCNLP approach could be used by decision makers based on the projected applicable conditions and the interrelationships between system uncertainties, risk probabilities, and economic objectives. 
